# The Evolution of Trust Within a Global Health Partnership With the Private Sector: An Inductive Framework

**DOI:** 10.34172/ijhpm.2021.14

**Published:** 2021-03-06

**Authors:** Sarah Christie, Teresa Chahine, Leslie A. Curry, Emily Cherlin, Erika L. Linnander

**Affiliations:** ^1^Global Health Leadership Initiative, Yale School of Public Health, New Haven, CT, USA.; ^2^Yale School of Management, New Haven, CT, USA.

**Keywords:** Global Health Partnerships, Public-Private Partnerships, Trust, Inductive Framework, Sub-Saharan Africa

## Abstract

**Background:** Public-private partnerships (PPPs) in global health are increasingly common to support sustainable development and strengthen health systems in low- and middle-income countries. Since the release of the Sustainable Development Goals (SDGs) in 2015 culminating in a discrete goal "to revitalize the global partnership for sustainable development," public health scholars have sought to understand what makes PPPs successful in different contexts. While trust has long been identified as a key component of successful strategic alliances in the private sector, less is known about how trust emerges between public- and private- sector partners, particularly in global health. Therefore, we investigated how trust between partners evolved in the context of Project Last Mile (PLM), a global health partnership that translates the business acumen of The Coca-Cola Company to strengthen public health systems across Africa.

**Methods:** This study draws upon secondary analysis of qualitative data generated as part of the longitudinal, mixed-methods evaluation of PLM across country settings. Seventy-seven interviews with a purposeful sample of key stakeholders were conducted in Mozambique, South Africa and eSwatini between August 2016 and July 2018. Trained qualitative interviewers followed a standard discussion guide, and audio-recorded interviews with participants’ consent. In this secondary analysis, we analyzed qualitative data to understand how trust between partners was cultivated across settings.

**Results:** We drew upon stakeholder experiences to inform an inductive framework for how trust develops over time. Our analysis revealed five domains that were foundational to building trust: (1) reputational context, (2) team composition, (3) tangible outputs, (4) shared values, and (5) effective communication.

**Conclusion:** The framework may be useful for private and public sector entities seeking to establish and sustain trust within their global health partnerships.

## Background

**Key Messages**

** Implications for policy makers**

- Building trust between partner organizations in global health partnerships with the private sector is foundational to the success of these partnerships, yet little is known about how trust is cultivated among stakeholders.
- We used stakeholder interviewers to understand how trust evolved in Project Last Mile (PLM), a public-private partnership (PPP) that leverages business expertise from The Coca-Cola Company to strengthen health systems in Africa.
- Five domains were conducive to building trust: (1) reputational context, (2) team composition, (3) tangible outputs, (4) shared values that extend beyond the outputs, and (5) effective communication through informal and formal mechanisms. We demonstrate how these five domains evolved over time.
- The resulting framework will be helpful for government agencies, development organizations, and private sector partners seeking to build trust for effective partnerships in global health.

** Implications for the public**

Public-private partnerships (PPPs) for global health are an integral part of the Sustainable Development Goals (SDGs) 2030, and building trust is important for effective partnership, yet little is known about how trust evolves in global health partnerships with multinational corporations. This study used qualitative data from Project Last Mile (PLM), a global health partnership with The Coca-Cola Company that aims to strengthen health systems across Africa, to understand how trust was developed in the context of PLM partnerships in eSwatini, Mozambique, and South Africa. Our analysis revealed five domains conducive to building trust within the partnership: (1) reputational context, (2) team composition, (3) tangible outputs, (4) shared values, and (5) effective communication. These findings can assist global health and development partners in understanding how trust can be cultivated and sustained with diverse partners for global good.

Public-private partnerships (PPPs) in global health are increasingly common mechanisms to support sustainable development, improve universal access to care, and strengthen health systems in low- and middle-income countries.^1-3^ As of 2019, the United States Agency for International Development (USAID) had built over 1800 partnerships with the private sector and such partnerships have proliferated across the donor landscape.^4^ Similarly, international development organizations including the United Nations have become increasingly interested in PPPs, culminating in the inclusion of a discrete Sustainable Development Goal (SDG) for 2030 “to revitalize the global partnership for sustainable development.”^5^

 In global health, partnerships are defined as a commitment to a common goal through the joint provision of complementary resources and expertise, and the joint sharing of risks involved.^6^ Illustrative examples include Gavi, the Vaccine Alliance,^7^ Merck for Mothers,^8^ and Project Last Mile (PLM).^9^ Since the release of the SDGs in 2015, public health scholars and practitioners have made strides in shaping our understanding of what makes PPPs successful in different contexts. For example, Gavi, the Vaccine Alliance, describes a partnership framework^10^ which highlights three drivers that add value to partnerships: trust, a clear governance mandate, and a diverse and inclusive network. Similarly Alonazi^11^ identified 5 critical factors in maintaining successful and sustainable PPPs including (1) trustworthiness, (2) technological capability, (3) patient-centeredness, (4) competence, and (5) flexibility.

 Inter-organizational network theory has long identified trust as one of the key components of a successful strategic alliance among corporations operating in the private sector,^12,13^ and trust is perhaps even more important in the realm of partnerships between public and private-sector organizations.^14-16^ Given the power imbalances that can occur in global health partnerships^17^ and potential for conflicts of interest,^18^ trust in the motivations and integrity of all parties involved in such partnerships is crucial to their long-term success.^14,15^ Nonetheless, a recent review by Kostyak et al^18^ confirms that public sector mistrust and unclear motives of the private sector are frequently cited as challenges for global health partnerships, as each partner organization must carefully consider how partners align with their mission, values, and policies.^19^ The issue of misalignment came to the fore with the dissolution of the partnership between the Global Fund and global beer producer, Heineken, due to the company’s perceived role in the proliferation of alcohol consumption in Africa, contributing to many of the public health issues that the partnership sought to address.^20,21^

 Despite the established role of trust in strategic alliances with the private sector, less is known about how trust is cultivated within PPPs for global health.^22,23^ Therefore, we investigated how trust evolved using longitudinal, qualitative data from PLM, a global health partnership that shares the technical expertise and business acumen of The Coca-Cola Company with public health systems across Africa. Since 2014, PLM has been supported by a Global Development Alliance that includes the Global Fund to Fight AIDS, TB and Malaria (the Global Fund), USAID, Bill & Melinda Gates Foundation, The Coca-Cola Company and its Foundation. The Global Health Leadership Initiative at the Yale School of Public Health has been monitoring and evaluating the partnership since its inception.^23^ We sought to understand how trust was cultivated in practice across sectors and settings, and to highlight key considerations in developing and strengthening mutual trust within global health partnerships, particularly with multi-national brands. We drew upon stakeholder experiences to inform a framework for how trust develops over time in this context. The framework may be useful for private and public sector entities seeking to establish and sustain trust within their global health partnerships.

## Methods

###  Study Settings

 This study includes data from three African countries in which PLM has been deeply engaged since 2016, as described below.^24^ Notably, we selected these three cases because they are exemplary of a longstanding, global PPP in practice, and because they provide rich and diverse contexts in which to understand how trust among partners evolved.

####  Project Last Mile in South Africa

 Since April 2016, PLM in South Africa has been funded by the USAID South Africa Mission to serve as a national strategic partner to the National Department of Health (NDoH) in support of its Central Chronic Medication Dispensing and Distribution (CCMDD) program.^25^ The CCMDD program aims to decongest facilities and provide stable patients with convenient pick-up points to collect medications for chronic diseases, including HIV and non-communicable diseases (eg, hypertension and diabetes). These pick-up points include fast lanes at participating health facilities, community-based adherence clubs, and external pick-up points co-located at private sector retailers and independent pharmacies.

####  Project Last Mile in Mozambique

 Since June 2016, PLM in Mozambique has been supported by the Global Fund to Fight AIDS, TB and Malaria (the Global Fund) to support the Central Medical Stores (Central de Medicamentos e Artigos Medico, CMAM) in implementing its Pharmaceutical Logistics Strategic Plan^26,27^ to transition its warehouse and distribution model from roughly 160 district and provincial stores to 30 strategically-placed intermediary warehouses.

####  Project Last Mile in the Kingdom of eSwatini

 In August 2017, with support from the Global Fund, PLM in eSwatini partnered with the Ministry of Health and its Health Promotion Unit as well as the National Emergency Response Council on HIV and AIDS to support a strategic marketing project to increase demand for HIV prevention services, including HIV testing, amongst adolescent girls and young women (AGYW).

###  Study Design

 This study draws upon qualitative data generated as part of a longitudinal, mixed methods evaluation of PLM’s work across country settings (as described previously).^25^ The design was grounded in participatory research principles.^25,28-30^ For example, the evaluation team worked with PLM project managers to identify measures of partnership success that were locally-relevant and feasible to track, to align timing of rounds of qualitative data collection with partnership milestones, and to identify and recruit interviewees who had deep experience with the partnership, including public sector officials, donors, and private sector partners.

 We used purposive sampling to identify 77 key stakeholders from the 3 countries who represent diverse partner perspectives (see Table), engaging additional stakeholders until we reached theoretical saturation in each round (ie, until subsequent interviews revealed no new information about the partnership).^31^ Between August 2016 and July 2018, we conducted in-person interviews in each country after the completion of discrete project phases. Individuals who could not participate in-person during our field visits were reached using a secure teleconferencing platform. Seven out of the 26 respondents in South Africa Phase II also participated in Phase I.

**Table:** Interview Participants

	**South Africa Phase I [August 2016] **	**South Africa Phase II [October 2017] **	**Mozambique Phase I [October 2017]**	**Kingdom of eSwatini [July 2018]**	**Total**
Public Health Sector^a^	8	7	8	7	30
NGO Support Partner	2	4	-	2	8
Private Sector Partner including Coca-Cola	0	6	4	6	16
Donor Partner	1	1	2	1	5
PLM Team	2	8	5	3	18
Total	13	26	19	19	77

Abbreviations: NGO, non-governmental organization; PLM, Project Last Mile. ^a^CMAM in Mozambique; NDoH in South Africa; Ministry of Health in eSwatini.

 In-depth interviewers were conducted by a trained qualitative interviewer following a standard discussion guide that included open-ended questions about the perceived strengths and benefits of the partnership, challenges and barriers to the partnership, as well as perspectives on the working relationship amongst the partners (Supplementary file 1).The discussion guide did not include specific questions or prompts to elicit reflections on trust between partners; however, this construct emerged as a salient code through the iterative coding process described below.

 All interviews were conducted and audio-recorded with participants’ consent. Audio recordings were professionally transcribed for analysis. This study was approved by the Yale Human Subjects Committee; CMAM in Mozambique; the NDoH in South Africa; and the Ministry of Health in Swaziland.

 For the parent study, three members of the research team independently coded each transcript, using the constant comparative method^32^ to generate a codebook that started with a priori codes based on evaluation goals, which was refined as new constructs emerged and reapplied to all transcripts once finalized **(**Supplementary file 2).^23,25^ The research team met regularly to compare and reconcile coding decisions through negotiated consensus.

 In this secondary analysis, we retrieved data that had been coded with the Trust & Motivation subcode to create a new dataset. Two new members of the research team independently coded and analyzed this dataset using an iterative approach to thematic analysis^33^ to inductively generate a more detailed codebook specific to this substudy **(**Supplementary file 3). We identified key constructs that emerged related to trust among partners, with attention to identifying negative or disconfirming information throughout thematic analysis. The analysis was managed using ATLAS.ti v7.

## Results

 Participants represented diverse perspectives from the various stakeholders of PLM. Nineteen participants were interviewed in Mozambique and eSwatini each, while 32 participants were interviewed from South Africa over 2 phases (Table).

###  Framework for Developing Trust

 Data analysis revealed five domains that were foundational to building trust, and are represented in the inductive framework shown in Figure:reputational context, team composition, tangible outputs, shared values, and effective communication. The arrows represent the evolution of trust in interrelated stages that unfold over time. Each domain is described below, with illustrative quotations.

**Figure:**
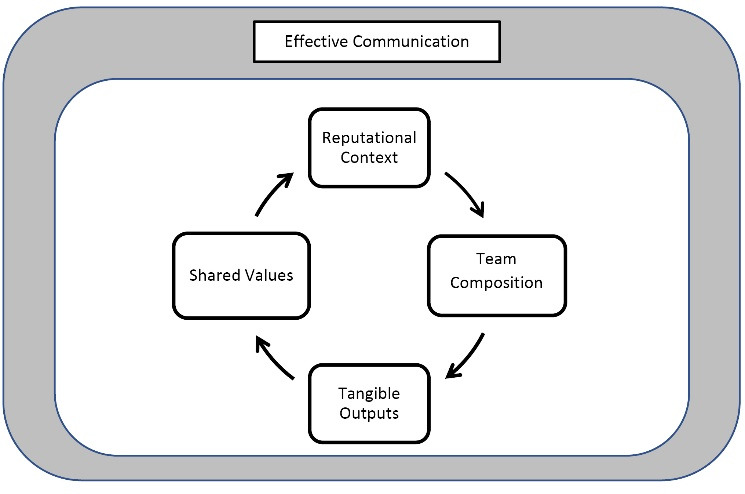
Framework for Developing Trust *Over Time* in PPPs in Global Health. Abbreviation: PPPs, public private partnerships.

###  Reputational Context

 The first domain of the framework requires developing a clear understanding of perceived reputations and motivations of the private and public sector partners, to inform subsequent trust building efforts. Importantly, organizational reputations as perceived by both the partners and the public varied across country settings and over time. For example, in South Africa, the association of PLM with Coca-Cola was regarded as a potential risk because of public mistrust of the food and beverage industry.

*“In South Africa, we can’t even say Coca Cola in the meetings. It’s looked upon very differently, as opposed to in Mozambique (CMAM). When you bring up Coca Cola, it’s a respectful thing. Coca Cola came to our meetings as well (in Mozambique) and added a lot of credence”* (PLM, Mozambique; ID 01).

 However, in Mozambique, Coca-Cola’s brand reputation was an asset that brought credibility to the project.

*“If government is working with Coke, this must be some kind of good project”* (Private Sector Partner, Mozambique; ID 12).

 In eSwatini, the association between Coca-Cola and health programming was difficult to convey to stakeholders within the public health sector early on *(‘Coca-Cola is related to soft drinks, not being healthy…it confuses people’) *(PLM, eSwatini; ID 03). As PLM staff worked deliberately to navigate brand perceptions with key stakeholders, initial skepticism dissipated. Over time, public sector representatives began to see the benefits of the partnership, such as learning successful marketing (‘*Coca-Cola is a brand known world-wide. If they say they have a strategy, you sit up and you want to learn’) *(Public Sector Partner, eSwatini; ID 04) and applying these principles to reach patients.

*“If we’re able to apply the principles as Coca-Cola have done. You’d want the target group when they see a certain sign in this facility, they’ll be assured of the package of services ….So, that’s what we anticipate, if we’re able to really tap on that. The people, the young, they will be assured that in the end, I’ll be accepted. I’ll get the services that meet my need” *(Public Sector Partner, eSwatini; ID 16).

 Others found that, with the Coca Cola connection, *‘you can open doors:’*

*“When there’s a commercial relationship between a company like Coke and an agency, you can open doors. That’s all it is. There’s nothing illicit. There’s nothing underhanded. The minute I send the email in the position of Marketing Director, people respond. It’s as simple as that” *(Private Sector Partner, eSwatini; ID 02).

 At the same time, some private sector views regarding public sector governance and administrative processes influenced the partnership dynamics. They reflected on the relative costs and risks among partners, expressing concern with contractual requirements and associated payment delays that slowed the project from getting *‘up and running.’*

*“Now the costs to us, the risk to us, is infinitely higher than the risk to them. We could have been up and running with this project three months ago if we just had that signature. Nothing’s changed. Our commitment to the project, our planning, our systems remain the same. The only thing that we haven’t got is the contract signed” *(Private Sector Partner, South Africa; ID 28).

 Amidst varying degrees of ambivalence between sectors, the introduction of a bridge, or an entity to navigate across sectors, was considered essential. As this participant from the NDoH reflected, bringing PLM on board helped to facilitate the integration of private sector expertise and address the historical distrust among sectors in order to achieve public health goals:

*“There’s always been a distrust between the public sector and the private sector. It was on that premise that I brought PLM on board. I had to see how to create systems and business models that work, whereby I can contract the private sector to render care for my patients, but still achieve my public health imperatives?... The unique position that PLM has brought is an understanding of how I can leverage off the efficiencies of the private sector, yet retain the characteristics that one would require of a public health approach” *(Public Sector Partner, South Africa; ID 10).

###  Team Composition 

 Building on this understanding of the reputational context and need for a bridge between sectors, PLM partners turned to developing a core project team, tending to both its composition and clarity on roles and expectations. In selecting Country Leads, the PLM team determined whether the person was a *‘fit’* and looked to see *‘positive chemistry’ *(PLM, South Africa; ID 19)among partners. PLM enlisted Country Leads who were well-regarded not only for their technical expertise but also for their adept interpersonal skills in navigating a complex landscape of partners and stakeholders. Participants observed that Country Leads were *‘very calm and very diplomatic,’ *(Private Sector Partner, Mozambique; ID 10) as well as ‘*just willing to be there and help us when we need the help’* (Public Sector Partner, South Africa; ID 14). Country Leads became embedded in the system as critical boundary spanners,^34^ trusted across sectors. One participant reflected on a Country Lead’s skill to *‘see where these two worlds meet and how they can jive together’* (Private Sector Partner, eSwatini; ID 13).

 PLM team members worked with partners to define individual roles with attention to engaging diverse and unique skills and perspectives. One partner noted the importance of defining roles before implementing tasks: *‘Each of the persons to play his role, and then we go’ *(Private Sector Partner, Mozambique; ID 09). Another reflected that donor-prescriptiveness is common, yet had not emerged in this project:

*“Things can get quite donor-prescriptive … I haven‘ t experienced any of that in this project. Everyone has come into their role and played their role and brought things to the table that other people didn‘ t have. Everyone has been able to work towards their skill and their resources that they‘ ve been able to add … there‘s always been an equal playing field … there hasn’t been this level of prescription” *(Private Sector Partner, eSwatini; ID13).

 Significant effort was invested in understanding and leveraging the assets and potential of the respective partners, and stakeholders described an openness to authentic collaboration. A private sector partner reflected on the absence of defensiveness, which cultivated mutual trust.

*“No one was putting up their defenses … It was very much collaborative back and forth, and a high level of trust … the level of genuine connection, concern, was good … It was just very much an open discussion, which was really nice” *(Private Sector Partner, eSwatini; ID 19).

###  Tangible Outputs

 Delivering concrete outputs was the *‘bedrock’* in unifying partners across sectors and catalyzing momentum. In each country setting, the value of the niche technical expertise afforded by PLM, such as route optimization, strategic marketing, or geomapping of client populations to improve health service delivery, grew more apparent over time. In Mozambique, the public sector was initially uncertain about the value of PLM, but once the first set of analytic reports and recommendations were shared, strong interest in the products emerged:

*“They need to prove themselves. They said they would do this, this and this…They had to go out to the provinces and do all this work. As soon as they made the first presentation from the first province on their route optimization, people said, ‘Oh, this is what we need. We really want this’” *(Donor Partner, Mozambique; ID 19).

 In all country settings, trust consolidated as PLM delivered on tangible workstreams. As teams developed ways of working, members reflected on the value of delivering outputs in solidifying trusting relationships.

*“The project started as being abstract because it was still under development. The relationship is improving … because it has been introduced in a number of forums now -beginning to understand exactly what we’re talking about when we say we have support to help us in communication, and it’s based on the expertise and the experience in the Coca-Cola area of communication. Because it’s beginning to take shape in terms of activities” *(Public Sector, eSwatini; ID 17).

 Further, partners prided themselves on prioritizing responsiveness (*‘we make sure queries are handled quickly… we are here to help’*) (Private Sector Partner, South Africa; ID 31). Shared commitment to the partnership was made visible through persistence through challenging circumstances (‘*they saw we were not walking away when it got hard’) *(PLM, Mozambique; ID 14). Through these interactions and in working together to deliver valued outputs over time, PLM built mutual trust in the partnership.

*“I’m getting really involved. I’m taking my time to be in the meetings, to share information … Now, these people need help. There are people in very far away communities which don’t have access to medicines, and somehow, we are helping them to get that access” *(Private Sector Partner, Mozambique; ID 10).

###  Shared Values

 As teams worked together to achieve various deliverables, partners developed an appreciation for complementary expertise (*‘everyone respected each other in terms of the stable we were in‘*) (PLM, Mozambique; ID 01). Additionally, recognition and respect for the diverse perspectives of the private and public sectors helped align on shared values. Finding common ground required the private sector to understand public sector constraints, including administrative processes and lengthier timelines; and the public sector to acknowledge the constraints of the private sector with respect to availability and alignment of their contributions with core business activities.

 All sectors were unified by a shared authentic concern for the patient, which allowed them to bridge institutional differences. Partners perceived the private sector’s motivation as altruistic (‘*they don’t do it for the money per se, they do it because it is the right thing to do’) *(PLM, South Africa; ID 11). Stakeholders from both the private sector and public sector reflected on the strong connection they felt to the partnership, and the shared perspective that it was making a difference in patients’ lives, by improving access to medications and services. *‘Everyone does have patients in mind … that resonated with the team quite effectively’ *(PLM, Mozambique; ID 1). Participants from both sectors pointed to genuine concern for patients as their core motivation for engaging in the project.

*“I’m excited about the fact that my patients do not have to spend so many hours at a clinic getting their medication. That allows them to spend more time at home [and] at work. That’s what I’m excited about. That’s my motivation…” *(Private Sector Partner, South Africa; ID 27).

*“That’s really my—my passion is to get to the patient in the end…”* (Public Sector Partner, South Africa; ID 14).

 Similar reflections were offered by stakeholders in eSwatini.

*“At first, it was very sobering, and then people began to open up and realize… what was really happening (with AGYW)… We‘re all in business, we‘re all professionals, but I really felt that they were bought-in, and that they really cared about it. It wasn‘ t just, okay, that‘s what we would expect, et. cetera. It was very human and very genuine… which I appreciated” *(Private Sector, eSwatini; ID 19).

###  Effective Communication

 Each of the domains of the trust-building process relied fundamentally on consistent, open, and timely communication. PLM supported such routine communication through formal supports and governance structures, as well as commitment to transparency and knowledge translation. In Mozambique, a regularly convened Steering Committee with participants from PLM, CMAM and Coca-Cola was considered ‘critical’ in implementing the project and keeping partners accountable (PLM, Mozambique; ID14). Similar feedback was provided by stakeholders in eSwatini, who participated in the Technical Working Group which was regularly convened for consultative feedback and inputs.

*“It’s been a smooth working relationship, because we not only meet for Project Last Mile, we have other avenues where we get to meet. We work together almost daily, so it’s not like, ‘Oh, it’s this thing.’ It’s normal” *(Public Sector Partner, eSwatini; ID 4).

 In South Africa, PLM was often referred to as ‘embedded’ within the NDoH structure and governance (Public Sector, South Africa; ID14). The PLM Country Leads’ presence in-country, both on an ongoing basis and at critical times, was seen as key to *‘make it work.’* The ability to have agile, real time meetings was described as a central feature of communications in all countries.

*“[x] spent a lot more time in -country just being on the ground and touching base with everyone at crucial times in the project. He really has spent the time here to make it work…Being flexible and being able to just be available just to have a quick pop -up meeting if someone’s available at the drop of a hat. That’s been crucial to the success of this project” *(PLM, Mozambique; ID 2).

 Team members reflected that communications were characterized by high levels of transparency around expectations, coupled with safety to speak candidly (“*everyone knew what was expected of them…If anything was out of line we had no problem saying, ‘That was out of line. Let’s move on’’ ’)*(PLM, Mozambique; ID 1).Reflecting on the partnership’s evolution over time, one participant noted strengthening of relationships that allowed for difficult conversations (‘*we can really discuss hot issues without killing each other’) *(NGO Support Partner, South Africa; ID 13). Open access to complete information was regarded as *“amazing*:*”*

*“ So it was amazing, because every stakeholder was there. And every time they came out with some results, we invited everyone to show them. And that was a key point, to get all the information, all the results. …they didn’t deny any information. They gave everything we asked for. They were very open” *(Donor Partner, Mozambique; ID 17).

 Informal supports for effective communications included the ability to translate across the jargon common in each sector, and speak the local language. One participant highlighted the importance of a PLM Country Lead who could ‘*in simple terms, explain the value add of CCMDD…articulating something into the language of who you are trying to get buy in*’ (PLM, South Africa; ID 19). One participant recounted a transformational shift from the private sector, which was initially reticent to engage with the public sector, until the PLM lead created ‘*a narrative’ *to help them understand the value proposition.

*“The project lead has understood from the private sector’s perspective their business model and just spent a lot of time getting his head around what our [CCMDD] business model has been. He’s managed to create a narrative or a discourse that had taken us from the private sector being fairly cautious about interacting with governments to a point where at the moment we’re having to slow down involvement of pick-up points…” *(Public Sector Partner, South Africa; ID 10).

 Stakeholders also reflected on the need for patience to translate expertise to the public sector, and described the necessity of authentic collaboration, rather than spoon-feeding skills or top-down expertise.

*“If you want to help, you must instruct people to do, not just spoon feeding. When we started saying, ‘Okay, we can’t do this, but we can help you on getting the results,’ then they started working. When we went through meeting more often to understand what needs to be done, then things started flowing”* (Private Sector Partner, Mozambique; ID 9).

 While there was acknowledgement that open and responsive communication created an environment conducive to building trust, it took significant time for the PLM team to understand the current reality, local dynamics and competing demands of in-country stakeholders, particularly around the initiatives that the partnership intended to support.

*“It takes so much longer than I think. Because I always forget how long it takes to build relationships… such that you’re with a cross-section of people to go radically alter the way that they do things” *(PLM, eSwatini; ID 1).

## Discussion

 Based on experiences of stakeholders across three country settings, we developed an evidence-informed framework for the development of trust in PPPs for global health. The five domains (reputational context, team composition, tangible outputs, shared values, and effective communication) are consistent with both the management literature on trust^35^ and the public health literature on building trust in partnerships,^22,36-38^ and extend this literature by exploring how trust is manifest in a PPP with a multinational brand.^2,35,39-42^ Our findings suggest that trust evolves over time as teams interact, outputs are delivered, and governance structures reinforce interpersonal interactions and shared achievements.

 First, the credibility of the country leads, coupled with sharing of data and expertise through repeated interactions between partners clarified and reinforced the positive intentions of PLM. This dynamic is consistent with theoretical literature on how positive expectations of the intentions of other parties are established and reinforced.^43-46^ Stakeholders appreciated ‘face-time’ with the PLM Country Leads, and acknowledged the extra effort and responsiveness that each afforded to the workstreams. Front line workers, particularly in South Africa, appreciated the willingness of country leads to visit sites to assess the current reality and work with local District Health Offices. These interactions were strengthened during regular and ad hoc touch points with multi-level stakeholders throughout the project.

 Second, delivering tangible outputs was viewed as foundational to trust; the deepening of trust then played a role in amplifying partners’ ability to collectively generate contributions toward their project goals. This finding coheres with other literature where trust acts as a more effective mechanism than contracts in facilitating the flow and application of knowledge,^41,47^ strengthening an organization’s ability to use new knowledge for demonstrable impact^48^ The growth of collaborative know-how reinforces partners’ trust by creating specialized knowledge and opportunities for further exchange, application, and diffusion.^47-50^ While this has been demonstrated in the management literature among the private sector, our study illustrates its application in a global health PPP context.

 Third, we found that effective communication through structural channels [ie, governance mechanisms],^42^ contributed to accountability and transparency in both eSwatini and Mozambique,^39^ reinforcing interpersonal interactions and providing a platform to share outputs. This further led to trust at an interorganizational level.^42^ Bump proposed a unified definition of governance consisting of three principles: accountability, participation and responsiveness,^51^ which were reflected in experiences of PLM stakeholders.

 Several limitations to the study must be noted. First, social desirability response bias may have occurred,^52^ in that participants may have vested interest in describing the partnership in a positive light. However, these results are based on interviews with multiple respondents, across settings and interviews were conducted by external evaluators who used established techniques^53,54^ to encourage participants to share both positive and negative experiences.^55^ Second, the PLM partnership reflects a novel partnership model with The Coca-Cola Company, and the extent to which our findings are generalizable and representative beyond this partnership is untested. Nevertheless, our themes were consistent across three diverse settings and distinct workstreams, and the framework is consistent with both the management and public health literature on how strategic alliances are formed, particularly in the private sector. Third, the parent study was not prospectively designed to describe or evaluate trust among partners, and we relied on secondary analysis of a subset of data identified through the use of a single Trust and Motivation subcode from the parent study. However, the emergence of the trust construct across settings in the parent study, and the richness of the data for this substudy, suggest the data are sufficiently robust to support our findings. Finally, our dataset did not allow us to assess public trust in the partnership from a community stakeholder perspective, which would be valuable as a future avenue of research.

## Conclusion

 As global health partnerships with the private sector have proliferated, there have been multiple calls to gather evidence on their impact and understand how partnerships navigate trust in this complex landscape to ensure accountability, particularly from the perspective of the public sector.^2,35,39,40^ This study presents a multi-faceted understanding of how trust evolves over time within PPPs for public health. The resulting domains can help diverse actors focus their efforts to cultivate trust over time within their global health partnerships, where intrinsic tensions between private and public sectors can hinder program implementation. Understanding perceptions of the private sector and specific brands is important for corporations and donors as a baseline to inform how the partnership will be perceived and taken up by the public sector. Recruiting the right people from the private sector – who are technically skilled, but also dynamic, authentic and culturally-competent – is essential to build trust with the public sector. Delivering results, sharing data, and establishing governance mechanisms assists to keep all partners aligned, informed and actively engaged in the partnership. Responsive communication sustains trust over time. Finally, the authentic and shared concern for patient benefit is the aligning value that cements trust in the partnership, and helps partners maintain focus when tensions between private and public sector ways of working arise. We anticipate that this study may be useful for innovators from both sectors seeking to create more effective partnerships for global good, and to evaluators of partnerships seeking to incorporate evidence-informed models of trust into their conceptual frameworks.

## Acknowledgements

 The authors thank the PLM team and their collaborators for sharing their perspectives and experiences during in-depth interviews. The authors also acknowledge Dr. Amy Wrzeniewski, Dr. Shahira Ahmed, Katherine Mertens, Mahrukh Zahid, and Diana Estefania Estrada Alamo for assistance in preparing this manuscript.

## Ethical issues

 The evaluation of PLM was approved by the Yale Human Subjects Committee; CMAM in Mozambique; the NDoH in South Africa; and the Ministry of Health in the Kingdom of eSwatini, formerly known as Swaziland.

## Competing interests

 Authors declare that they have no competing interests.

## Authors’ contributions

 SC and EC analyzed the data with supervision from LAC; SC and TC drafted the original manuscript with substantial revisions from LAC and ELL; all authors have read the manuscript, provided critical review, and approved the final manuscript.

## Funding

 The PLM activities in Mozambique and the Kingdom of eSwatini are funded by the Global Fund to Fight AIDS, Tuberculosis and Malaria; activities in South Africa are funded by USAID. The evaluation by the Yale Global Health Leadership Initiative is funded by USAID and The Coca-Cola Company.

## Supplementary files

Supplementary file 1. Discussion Guides.

Supplementary file 2. PLM Code Book - November 13, 2018.

Supplementary file 3. Trust Code Book - September 10, 2018.
