# Supplementary material for: The Evolution of Trust Within a Global Health Partnership With the Private Sector: An Inductive Framework
Source: Int J Health Policy Manag. 2021 Mar 6;11(7):1140–7. doi: 10.34172/ijhpm.2021.14 (PMC9808177; doi:10.34172/ijhpm.2021.14)
Supplement: Supplementary file 1 — Discussion Guides. [file ijhpm-11-1140-s001.pdf]

Article Title: The evolution of trust within a global health partnership with the private sector: An inductive framework

Journal name: International Journal of Health Policy and Management (IJHPM)

Authors: Christie S[1], Chahine T[2], Curry LA[1,2], Cherlin EJ[1], Linnander EL[1]

[1]Global Health Leadership Initiative, Yale School of Public Health

[2]Yale School of Management

Authors' Information:

1. **Sarah Christie, MPH (Corresponding Author)**
Primary Affiliation: Global Health Leadership Initiative, Yale School of Public Health, New Haven, CT USA
Email: sarah.christie@yale.edu
ORCID: 0000-0003-3907-2856
Phone: 475-439-4660
Address: 100 Church Street South, Suite A199, New Haven, CT, USA

2. Teresa Chahine, MSc, ScD
Primary Affiliation: Yale School of Management, New Haven, CT USA
Email: teresa.chahine@yale.edu

3. Leslie Curry, MPH, PhD
Primary Affiliation: Global Health Leadership Initiative, Yale School of Public Health, New Haven, CT USA
Secondary Affiliation: Yale School of Management, New Haven, CT USA
Email: leslie.curry@yale.edu

4. Emily Cherlin, MSW, PhD
Primary Affiliation: Global Health Leadership Initiative, Yale School of Public Health, New Haven, CT USA
Email: emily.cherlin@yale.edu

5. Erika Linnander, MBA, MPH;
Primary Affiliation: Global Health Leadership Initiative, Yale School of Public Health, New Haven, CT USA
Email: erika.linnander@yale.edu

Supplementary file 1. Discussion Guides

Project Last Mile South Africa (Phase I)
Semi-Structured Interview Guide

Our team at Yale is documenting the Project Last Mile initiative, or PLM, in a number of countries, including South Africa. As you may know, PLM is a partnership to bring private sector expertise to public sector health systems. We are interested in learning about challenges and successes you are facing with this project. *We would like your permission to record this interview. This lets us listen carefully to you rather than taking notes and we will accurately capture our conversation. All information will be kept strictly confidential and no identifying information about you or your organization is included on the transcript. Digital files with audio-recorded material will be deleted as soon as the transcripts have been reviewed for accuracy. If at any point you would like me to turn off the recorder, please let me know. You are free to decline to participate, to end our interview at any time for any reason, or to choose to skip any question.*

1. Please describe your role.
    a. How long have you worked in this position?
    b. Please describe your involvement in CCMDD
    c. Please describe your involvement with Project Last Mile

2. Please describe the collaboration between the National Department of Health and Project Last Mile.
    a. Can you talk a little bit about how it has been going so far?
    b. How has the collaboration changed or evolved over time?

3. What has been going well so far?
    a. How did these successes come about?
    b. Have the changes been sustained over time?

4. Can you talk about any risks, setbacks, or struggles so far?
    a. How have you worked through these challenges?

5. How would you describe the relationships you have developed as part of PLM's involvement? (note to interviewer: use lots of probes here)

6. Is there anything I haven't asked you that would be helpful for us in understanding PLM and the CCMDD initiative?

At each step along the interview, the interviewer should **follow the story and ask questions to help us understand the story.** For instance, when asked about the initiative, the respondent might then say "We put a team together" to which the interviewer might ask "Who put the team together? Who was on it? Why were these people included? What were your hopes and concerns when you found out you were on it?"

General probes:

- Can you tell me more about that?
- What did you mean when you said "___________"?

Content Probes: Key quantitative metrics (impact indicators)

- Proportion of public health establishments where CCMDD programme is implemented
- # of patients enrolled in CCMDD
- # of patients picking up medication from external pick-up points
- # external pick-up-points appointed for CCMDD programme
- Average patient wait time
- Patient satisfaction

Project Last Mile Mozambique
Semi-Structured Interview Guide

Our team at Yale is documenting the Project Last Mile initiative, or PLM, in a number of countries, including Mozambique. As you know, PLM is a partnership to bring private sector expertise to public sector health systems. We are interested in learning about challenges and successes you are facing with this project. Our goal is to capture lessons learned to help improve the project in Mozambique and to share with other PLM projects. The interview questions are high-level, open-ended questions that invite you to reflect on your experience with the project so far.

We would like your permission to record this interview. This lets us listen carefully to you rather than taking notes and we will accurately capture our conversation. All information will be kept strictly confidential and no identifying information about you or your organization is included on the transcript. Digital files with audio-recorded material will be deleted as soon as the transcripts have been reviewed for accuracy. If at any point you would like me to turn off the recorder, please let me know. You are free to decline to participate, to end our interview at any time for any reason, or to choose to skip any question.

1. Please describe your role.
    a. How long have you worked in this position?
    b. Please describe your involvement with Project Last Mile

2. Please describe the collaboration between your organization and Project Last Mile
    a. How it has been going so far?
    b. How have things changed since the start?
    c. Have there been changes in your business practices or ways of working since the start of PLM? If so, can you share some specific examples? (Note: especially relevant for CMAM and implementation partners)

3. What has been going well so far?
    a. How did these successes come about?
    b. Have the changes been sustained over time?
    c. Have things been different than what you expected?

4. Can you talk about any setbacks, or struggles so far?
    a. How have you worked through these challenges?
    b. Have things been different than what you expected?

5. How would you describe the relationships you have developed through PLM? (note to interviewer: use lots of probes here)

6. How has the communication between the different supply chain partners been going?
    a. If it has changed, can you give an example?

7. Is there anything I haven't asked you that would be helpful for us in understanding PLM?

At each step along the interview, the interviewer should **follow the story and ask questions to help us understand the story.** For instance, when asked about the initiative, the respondent might then say "We put a team together" to which the interviewer might ask "Who put the team together? Who was on it? Why were these people included? What were your hopes and concerns when you found out you were on it?"

General probes:

- Can you tell me more about that?
- What did you mean when you said "___________"?

Content Probes:

Key project activities

CCS's fleet manager worked with PLM to plan for the route optimization pilot in Tete province, including preparations with a Coca-Cola driver.

- Warehouse location assessment
- Route-to-Market mapping and plan
- Route-to-market business case (cost, benefit, targets, requirements)
- Facilities reached in the pilot region
- List of potential suppliers for outsourced distribution and a supplier evaluation framework
- Benchmark cost data and estimated cost for the proposed RTM model(s), including key targets/ metrics based on Sabco experience
- Supplier performance and contract management process document (including any tools developed for CMAM or its suppliers)
- Performance management objectives, targets and high level review process for logistics management roles at Intermediary Warehouse level
- Relevant training materials developed for logistics staff at intermediary warehouses

Key quantitative metrics (impact indicators)

- Cost efficiency
- Delivery intervals to health centers
- Stock-outs of ARVs, vaccines, and syringes
- Distribution cost per kilometer
- % of Intermediary Warehouses with job profiles and job descriptions reviewed and updated
- % of Intermediary warehouse roles that have new performance management objectives and learning pathways set

Project Last Mile South Africa (Phase II)
Semi-Structured Interview Guide

Our team at Yale is documenting Project Last Mile in several countries, including South Africa. As you know, PLM is a partnership to bring private sector expertise to public sector health systems. Like with our first interviews in August 2016, we are interested in learning about challenges and successes you are facing with this project. Our goal is to document project progress over the past year, as well to as to capture lessons learned to help improve the project going forward and to share with other PLM projects. The interview questions are high-level, open-ended questions that invite you to reflect on your experience with PLM so far.

We would like your permission to record this interview. This lets us listen carefully to you rather than taking notes and we will accurately capture our conversation. All information will be kept strictly confidential and no identifying information about you or your organization is included on the transcript. Digital files with audio-recorded material will be deleted as soon as the transcripts have been reviewed for accuracy. If at any point you would like me to turn off the recorder, please let me know. You are free to decline to participate, to end our interview at any time for any reason, or to choose to skip any question.

1. Please describe your role in your organization.
    a. How long have you worked in this position?
    b. And what is your role with PLM

2. How it has been going over the past year?
    a. What has been going well? *(probe for concrete examples, and for details on each)*
    b. What about things that have not gone so well? *(probe for concrete examples and for details on each; especially how they have addressed the challenges)*
    c. Have there been changes in your business practices or ways of working in the past year? If so, can you share some specific examples? (Note: especially relevant for National Department of Health and implementation partners)

3. Has the project been different than you expected and if so can you talk about that?

4. How would you describe the relationships you have developed through PLM? *(probe for concrete examples)*

5. How has the communication between the different CCMDD stakeholders been going?
    a. If it has changed, can you give an example?

6. Is there anything I haven't asked you that would be helpful for us in understanding PLM and CCMDD?

At each step along the interview, the interviewer should **follow the story and ask questions to help us understand the story.** For instance, when asked about the initiative, the respondent might then say "We put a team together" to which the interviewer might ask "Who put the team together? Who was on it? Why were these people included? What were your hopes and concerns when you found out you were on it?"

General probes:

- Can you tell me more about that?
- What did you mean when you said "___________"?

Content Probes:
Key project activities

Geomapping of priority districts

Detailed mapping of 33 PEPFAR/NHI districts (current PuP facilities and Tier 1 PuP outlets, relative to population and burden of disease,
Opportunity mapping for deployment of PuPs
Provision of all captured data from contracted PuPs to NDoH central data hub

Route to market planning and support

Make recommendations for the implementation of detailed route-to-market options for CCMDD expansion
Co-develop CCMDD toolbox for health facilities and PuPs to ensure consistency of PuP standards
Support design of customized plans for districts, with province-level support
Support design of customized plans for service providers and private-sector partners, with NDOH-level support.

Private sector management

Test Picture of Success (PoS) plan and customize for districts
Test innovation options offered within the private sector
Support NDoH to develop contracts for private sector partners
Determine economies of scale for private sector partners and address barriers to PuP growth

Execution support for roll-out

Guide service providers on appropriate PuP options for designated territories
Support integration of CCMDD data and processes into Visual Analytics Network (VAN)
Support NDOH and partners to ensure CCMDD program standards are universally applied and that the CCMDD toolbox is embedded in the execution process

Key quantitative metrics (impact indicators)

Number of registered pick up points (nationally, per district)
Number of patients enrolled (nationally, per district)
Number of facilities enrolled (nationally, per district)

Project Last Mile Swaziland {eSwatini}
Semi-Structured Interview Guide

Purpose: The interviews to be conducted in July 2018 are being held to understand implementation of Project Last Mile in Swaziland [eSwatini], as well as highlight the strengths and challenges that have influenced the work at hand. Our goal is to understand lessons learned in Swaziland [eSwatini] and to share these with other similar projects that may take root in Africa.

As you know, PLM is a partnership to bring private sector expertise from the Coca-Cola system to the public health sector and this is the first demand creation project for the partnership, which will target young women and girls. We are also interested in learning about possible shortcomings of Project Last Mile, opportunities for improvement, and success stories. The interview questions are open-ended and invite you to reflect on your experience with the project since it was launched in August 2017.

We would like your permission to record this interview. This lets us listen carefully to you rather than taking notes and ensures that we will accurately capture our conversation. All information will be kept strictly confidential and no identifying information about you or your organization is included on the transcript. Digital files with audio-recorded material will be deleted as soon as the transcripts have been reviewed for accuracy. If at any point you would like me to turn off the recorder, please let me know. You are free to decline to participate, to end our interview at any time for any reason, or to choose to skip any question.

Discussion Points:

1. Please describe your role in your organization.
    a. How long have you worked in this position?
    b. And what is your role with Project Last Mile?

2. How has the project been going in the past year?
    a. What has been going well?
        i. Can you provide some examples of these strengths or successes? *(probe for concrete examples and for details on each)*
    b. What are some things that have not gone so well? Any setbacks, or challenges so far?
        i. How were you able to address these challenges? *(probe for concrete examples; especially how they have addressed the challenges)*
    c. Has the project been different than you expected and if so can you talk about that?

3. Have there been any changes in the way demand creation or health promotion have worked since Project Last Mile got involved? If so, how?

4. How would you describe the working relationships you have developed through PLM? *(probe for concrete examples)*

5. How has the communication between the team and different stakeholders been going? What has worked and what hasn't?

6. Is there anything I haven't asked you that would be helpful for us in understanding PLM in Swaziland [eSwatini]?

NOTE: At each step along the interview, the interviewer should **follow the story and ask questions to help us understand the story.**

For instance, when asked about the engagement, the respondent might say *"We put a working group together"* to which the interviewer might ask *"Who put the team together? Who was on it? Why were these people included? What were your hopes for what the working group would achieve?"*

General probes:

- Can you tell me more about that?
- Help me understand what you meant by "___________?

Content Probes:
Key project activities

Capability Assessment/Alignment [particularly important for Coca-Cola Marketing group]

- How has your experience with the Coca-Cola system assisted your engagement in PLM in Swaziland [eSwatini]? What expertise have been particularly valuable or useful thus far?
- How have private sector inputs been regarded by the public sector?

Stakeholder Engagement & Communication: There are many government, NGO, academic, and donor partners engaged in Swaziland [eSwatini] in the demand creation space.

- How have you been able to engage with the various stakeholders in this landscape?
- How would you describe the stakeholder relationships that have developed through PLM?
  - Which stakeholders have been supportive or Champions of PLM? Have any been problematic? If so, how?
- How has the communication structure between PLM and the stakeholders been going?
  - How is the Working Group functioning? Are they able to meet regularly and efficiently?

Demand Creation and Organization Capacity:

- How was working with the Research Agency to drill down on possible targets for intervention? How were findings conveyed from that work? Did you find them accurate and appropriate for demand creation in eSwatini? Please explain
- How are plans coming together for developing the media strategy with the Creative Agency? How has this process worked?
- How is the pilot media strategy emerging for Manzini? How receptive is the District to the activities that are being proposed?
- How do you think the demand creation and health promotion capabilities will change -if at all- after Project Last Mile? How would these efforts be sustained? What is needed to drive and continue this change?

Key quantitative metrics (impact indicators) -TBD

- What impact do you think this work will have on young women and girls in eSwatini? On youth in general? On the Manzini community?
