# Supplementary material for: The Evolution of Trust Within a Global Health Partnership With the Private Sector: An Inductive Framework
Source: Int J Health Policy Manag. 2021 Mar 6;11(7):1140–7. doi: 10.34172/ijhpm.2021.14 (PMC9808177; doi:10.34172/ijhpm.2021.14)
Supplement: Supplementary file 2 — PLM Code Book - November 13, 2018. [file ijhpm-11-1140-s002.pdf]

Article Title: The evolution of trust within a global health partnership with the private sector: An inductive framework

Journal name: International Journal of Health Policy and Management (IJHPM)

Authors: Christie S[1], Chahine T[2], Curry LA[1,2], Cherlin EJ[1], Linnander EL[1]

[1]Global Health Leadership Initiative, Yale School of Public Health

[2]Yale School of Management

Authors' Information:

1. **Sarah Christie, MPH (Corresponding Author)**
Primary Affiliation: Global Health Leadership Initiative, Yale School of Public Health, New Haven, CT USA
Email: sarah.christie@yale.edu
ORCID: 0000-0003-3907-2856
Phone: 475-439-4660
Address: 100 Church Street South, Suite A199, New Haven, CT, USA

2. Teresa Chahine, MSc, ScD
Primary Affiliation: Yale School of Management, New Haven, CT USA
Email: teresa.chahine@yale.edu

3. Leslie Curry, MPH, PhD
Primary Affiliation: Global Health Leadership Initiative, Yale School of Public Health, New Haven, CT USA
Secondary Affiliation: Yale School of Management, New Haven, CT USA
Email: leslie.curry@yale.edu

4. Emily Cherlin, MSW, PhD
Primary Affiliation: Global Health Leadership Initiative, Yale School of Public Health, New Haven, CT USA
Email: emily.cherlin@yale.edu

5. Erika Linnander, MBA, MPH;
Primary Affiliation: Global Health Leadership Initiative, Yale School of Public Health, New Haven, CT USA
Email: erika.linnander@yale.edu

Supplementary file 2: PLM Code Book- November 13, 2018

PLM Code Book—11.13.2018

Note: Do not use any codes with a "retired" prefix.

Code Name	Definition
**100. Context**	
101. Context	Enabling/disabling regulatory, legal and political factors; policy landscape; country context and culture; politics
102. Context – Gender/Social Norms	Refers to the social and cultural context as it pertains to gender equity, societal norms, vulnerability, cultural mores, traditions, power imbalance [that may influence demand creation but not necessarily]
**200. PLM Process**	
201. Knowledge transfer	Between Coke/PLM and other partners, sharing expertise, capacity building; actual transfer of knowledge or capabilities. Excludes private sector inputs
202. Private sector inputs_Technical	Technical expertise that PLM/Coke is providing to public sector partners, such as tools, data, analytic strategies, soft inputs, etc. Excludes marketing and communication inputs
202.a. Private sector inputs_Marketing&Comm	Marketing and communication inputs being provided by PLM/Coke to public sector partners. Includes assistance with branding, franchising, market research, creative design, demand creation, etc.
202.b. Private sector inputs_Expectations	Expectations about what the private sector/Coke will bring in terms of technical inputs, training, innovations, marketing, capacity building, tools, software, etc. that may or may not be met.
203. Adaptation/flexibility	Adaptations/shifts in response to circumstances, examples of how something was adapted to fit circumstances, context
204. Networks	Boundary spanners, active use of pre-existing relationships to make connections, development and dynamics of relationships that contribute to the work
205. Assessing and aligning "fit" between PLM and external partners	Assessment of the landscape, and planning for how PLM is/will be involved; Coordinating and finding complementary roles, complementary expertise; addressing role conflict/overlap and competing demands/priorities amongst partners; relationship-building and stakeholder engagement. Can also include aligning with donor priorities.
206. Process	Description of processes through which PLM contributes, the tasks and work done by PLM. The steps PLM has followed to reach their current involvement. Might include knowledge transfer, but not necessarily.
207. Data sources and evaluations	Any metrics or data that has been used in evaluating different components of PLM
208. Historical explanations	Explanations for how partnerships, processes, other aspects came to be.

209. Impact/sustainability	References to long-term results or impact of PLM project, including sustainability of the project after PLM implementation. Could include discussion of factors that may influence long-term results and sustainability.
210. Boundary spanner	Refers to a particular person in the PLM partnership who is able to communicate across public and private sectors
211. Governance	Refers to PLM project oversight, roles and responsibilities, planning and deadlines; also can refer to how PLM is governed by external structures and situated within organization
212. Route Optimization	Refers to PLM workstreams related to optimizing routes for distribution, including geomapping, supply chain logistics, related software and tools
213. Capacity Building	Refers to PLM workstreams related to specific capacity building, such developing organization structures, job descriptions, and performance management processes for the public sector
214. Outsourced Distribution	Refers to PLM workstreams related to facilitating and negotiating outsourced distribution
215. Demand Creation	Refers to PLM workstreams related to facilitating demand creation, including market segmentation and research, creative agency development, strategic communications, organization development, messaging
215a. Demand Creation – Research	Refers to the process of research to inform messaging and communication strategy, selection of the agency, can refer to quantitative or qualitative data collection and related procedures, research findings, receptivity to research findings
215b. Demand Creation – Communication Strategy	Refers to the process of creating and developing the communication strategy, selection of the agency, can refer to how the research findings informed the communication strategy, how the communication strategy will be rolled out (media placement, etc.), the message and media for the communication strategy, receptivity to the proposed strategy
215c. Demand Creation – Implementation	Refers to the process of putting the communication strategy into practice at the health facilities, how it will be operationalized, the sell-in for the health facilities, how it will be integrated into existing initiatives with existing personnel, training and capacity building, site selection
216. Innovation	Refers to innovation or lack thereof; when a respondent refers to something as a new way of doing things or explicit reference to an innovation that was contributed by PLM. Can also refer to expectations for innovation and whether the partnership met that expectation (and how).

**300. Influences on PLM process**	
301. Inter-sectoral communication	Communication across organizations/partners, such as between ministries of health and private sector partners. Excluded: communication within a single unit (department, level of system, or organization).
302. Communication within units	Communication within a department, level of the system, or organization.
303. Organizational culture	Descriptions of learning and problem-solving, ways of working together, commitment to organization, team work.
304. Buy-in and ownership	Extent to which individuals at different levels of the system feel ownership and want to support or be a part of PLM or PLM-supported programs, part of team.
305. Trust and motivation-for partnerships	Feelings of trust (or lack thereof) between partners, in the development of relationships and partnership. Also can indicate alignment on good will, public benefit or lack thereof (i.e., potential for conflict of interest).
306. Momentum	References to the pace at which PLM's work has moved; ebbs and flows in progress.
307. Funding/Resources	Refers to availability of financial/monetary resources as well as human resources.
308. External communications and advocacy	Refers to communication about and dissemination of PLM's work to organizations and stakeholders outside of the partnership
309. Bureaucracy and Contractual Considerations	Refers to contractual issues, bureaucratic processes, 'red tape', regulatory requirements and how they affect the partnership
**900. Overarching codes**	
901. Change	Any changes as a result of PLM's involvement; Changes to CCMDD; To be applied in addition to whatever changed (communication, organizational culture, etc.)
902. Barriers	Anything that has inhibited PLM, partnerships, CCMDD. To be applied in addition to whatever the barrier was (communication, organizational culture, etc.)
903. Facilitators	Anything that has facilitated PLM, partnerships, CCMDD, helped in overcoming challenges. To be applied in addition to whatever the facilitator was (communication, organizational culture, etc.)
904. Great quote	Quotations that are particularly illustrative of particular themes.
905. Successes	Anything described as a successful outcome of PLM. May or may not also be coded as change.
906. Feedback and Recommendations	Information that might be useful to provide as feedback to partners (delivery team, PMO, steering/working committees). Could be double-coded with a code that is specific to the recommendation, but not necessary.
