# Supplementary material for: The Evolution of Trust Within a Global Health Partnership With the Private Sector: An Inductive Framework
Source: Int J Health Policy Manag. 2021 Mar 6;11(7):1140–7. doi: 10.34172/ijhpm.2021.14 (PMC9808177; doi:10.34172/ijhpm.2021.14)
Supplement: Supplementary file 3 — Trust Code Book - September 10, 2018. [file ijhpm-11-1140-s003.pdf]

Article Title: The evolution of trust within a global health partnership with the private sector: An inductive framework

Journal name: International Journal of Health Policy and Management (IJHPM)

Authors: Christie S[1], Chahine T[2], Curry LA[1,2], Cherlin EJ[1], Linnander EL[1]

[1]Global Health Leadership Initiative, Yale School of Public Health

[2]Yale School of Management

Authors' Information:

1. Sarah Christie, MPH (Corresponding Author)
Primary Affiliation: Global Health Leadership Initiative, Yale School of Public Health, New Haven, CT USA
Email: sarah.christie@yale.edu
ORCID: 0000-0003-3907-2856
Phone: 475-439-4660
Address: 100 Church Street South, Suite A199, New Haven, CT, USA

2. Teresa Chahine, MSc, ScD
Primary Affiliation: Yale School of Management, New Haven, CT USA
Email: teresa.chahine@yale.edu

3. Leslie Curry, MPH, PhD
Primary Affiliation: Global Health Leadership Initiative, Yale School of Public Health, New Haven, CT USA
Secondary Affiliation: Yale School of Management, New Haven, CT USA
Email: leslie.curry@yale.edu

4. Emily Cherlin, MSW, PhD
Primary Affiliation: Global Health Leadership Initiative, Yale School of Public Health, New Haven, CT USA
Email: emily.cherlin@yale.edu

5. Erika Linnander, MBA, MPH;
Primary Affiliation: Global Health Leadership Initiative, Yale School of Public Health, New Haven, CT USA
Email: erika.linnander@yale.edu

Supplementary file 3: Trust Code Book – September 10, 2018

Appendix 3. Trust Code Book- 9.10.2018

PLM Code Book

Secondary Analysis on Trust

This code cook is to guide secondary analyses related to trust. It was built from review of codes 305 (trust and motivation for partnerships) and 304 (buy in an ownership). The goal was to develop a more detailed and nuanced understanding of the phenomenon of trust. A central theme that emerged from review of these codes was that trust is manifest as a continuum that evolved over time and across levels of the hierarchy. Trust matters at different levels and different points in time; partners have to be seen as trusted and credible orgs, GF, Gates, Coke; sometimes need trust at the high organizational levels and others - trust is key at the interpersonal front line.

**Code.**	**Description of Code**	**Example of Illustrative Quote**
730.Making and keeping promises	When partners make promises, commitments; were they honored/not and implications; putting in face-time; resources; meeting and managing expectations.	*The areas where we see the involvement, the direct involvement, is performing at a much higher growth rate than the others where the provinces are left to their own devices. They play very a pivotal role in guiding the districts on where to focus in identifying the pickup points. From that perspective, I think they're strategically well-placed in the role that they are playing there. We can see. We can see the positive outputs.* [SA Phase I]
731.Trust develops over time	Observations about how trust emerged over time; tipping points; when trust began	*I think CMAM was skeptical perhaps. I think CMAM was critical in the beginning, too. Then they realized that, oh no, I's serious—it's taking place. It's taking form. Then everything began to accelerate. Today I was super happy with the presentation they did. Yeah. PLM, they found their speed. They're good. We are really happy about what they are doing.* [Moz]
733. Natural/ecological fit (individual fit for the task at hand)	Refers to the unique or intrinsic attributes/ skills of a person or organization that are needed to achieve shared goal; can include personality, chemistry, diplomacy; mutual respect, including front line expertise; team dynamic; demonstrate respect by giving autonomy	*[The Country Lead] also gave a lot of value to the work because of his personality, his way of handling the staff, because the first steering committee that we had, people are very defensive that there was very tense in the meeting because they want results fast. They (CMAM) want visible things, and then they don't evaluate the work that it requires. So that first meeting, they were like, "No, this is tough. We want to see results. We want that." Then he's very calm and very diplomatic, and he managed to establish a very good relationship with them, and now they are happy.* (Moz)

734. Perspective taking	Refers to examples of members of one organization trying to understand the constraints, goals, priorities of another; also can refer to someone from public sector thinking 'outside the box' in line with private sector, or vice versa. Can also include 'team approach' – thinking outside of the silo, team perspective.	*I didn't know CCMDD from a bar of soap. I had to learn before I went into the district to talk to them about it, to the facilities, and stuff like that. As Pharmacy Direct, like I said previously, they've been predominantly private business. They haven't work with the government sector before. Having worked with government before, I had to listen- government works differently. You don't expect that government will give you facts or details because they don't have (them). It's a slow painful journey to getting them to understand that, but it is a journey that we have to take together, holding hands. Your expectations are there. They are there. You need to take them away. It's been very difficult. You've got bosses that expect this and this and that, or they like, "They haven't paid, and they haven't done this. What are you doing? Blah, blah, blah."*<br><br>*We work with government, guys. We're private business where they know they need to pay within 30 days. There's going to be challenges. What makes me happy is that, yes, we are a private business, and, ultimately, private is what we need to realize, but this program is what is going to make South Africa proud. As a service provider, as Pharmacy Direct, you should be proud that you are involved in something like this. We worry about margins, profit margins. We worry about this and that, how we're going to pay our self, but look at the bigger picture. (SA Phase II)*
735. Reciprocal gain	Each organization both gives and gains (Coke gives expertise and gains sense of 'doing good') – positive tension; can be altruism.	*Yeah, because we all know the same—our aim was the same. Our mission is the same. We all wanted to make sure that we establish a pickup point to support the patient. The patient being the core focus. Once everybody is on that footing and understands that, and is in the same playing field, I mean, we just rode the program. There was no more issues. When there was an issue, we sorted it out immediately. The relationship is fantastic, if you ask me. I mean, we pick up the phone and just chat with each other. It's not like we're different NGOs. We're in a team. We're in one team.* (SA Phase II).
736. Process of alignment of goals	Recognizing that partners exist within reality, people want to do good AND they need to follow their business imperative; also can refer to the tension of alignment between private:public sector and/resources, but also how alignment is sustained, and those tensions resolved.	*First, the private sector is very, very aware of the challenges government faces around the elements of funding. It's not just in the health sector; it's in everything. It's in education. It's just every aspect is short of money. They appreciate that, and I think if it was the only source of business, they would be extremely aggravated, but it's part of the corporate social responsibility with an economic benefit. The fact that we pay them the nine rand thirty seven, which is at today's exchange rate is about thirty five cents, US cents, for a parcel—they don't do it for the money per se, they do it because it is the right thing to do.* (SA Phase II)

737. Perceived objectivity (of partners, of data)	Coke not perceived to have COI or baggage; using performance-based, data driven lens vs politics	*One of the reasons are they're coming in cold, right? There's no baggage. They just understand that they are here to fulfill a role, and the way in which they are presenting their findings gives it a totally different perception. I think this is what the users are appreciating at that level. If they understand the challenges, they understand the complications, but in visualizing their challenges and how to address that, it's a total mind shift which the users are really appreciating*. -SA Phase I<br><br>*PLM, just through our resources are highly effective and inefficient. In fact, NDOH just said, "PLM is just efficient and effective. We just get stuff done." I thought that was pretty cool, because really, at the end of the day, we run businesses. We know how to get stuff done.*<br><br>*We've gotta be cognizant of the internal politics, et cetera, but we have a job to do. We have a frustrated audience, which are the nurses and clinicians that are looking for leadership and guidance. I think if we can help the department deliver that they will be in much better positions to grow their whole CCMDD strategy.*<br><br>*At the end of the day too, we're not a threat to anybody at the Department of Health, because we're not employees nor are we doing this for personal gain or political gain. We are a good solid partner with good knowledge, but we're not a threat.* – SA Phase I
738. Country cultural/ social context	Adapt approach to context (SA cannot say Coke, MZ = prestige); can also include when country context /government reputation that contributes to trust	*"In South Africa we can't even say Coca Cola in the meetings because of the industry that we were to the Ministry of Health. In South Africa it's looked upon very differently as opposed to CMAM. When you bring up Coca Cola, it's a respectful thing. Coca Cola came to our meetings as well and added a lot of credence."* -Moz
739. Communication	Ways in which communication brokered trust; aspects of communication that built trust – transparency; data sharing; routine meetings or regular communications; language nuance and compatibility; boundary spanning; coordination.	*I think the most significant elements of the partnership is that PLM has the ability to cut across all NGOs, all service providers, and all functions within the National Department of Health, which gives us a really good insight at a holistic level and a high level around all the elements that are involved in the pick-up points, adherence groups, etc. The biggest advantage that PLM has right now is the potential to be able to speak to multiple stakeholders, but also share information received from multiple stakeholders and then to consolidate that into a single message. The other big element, of course, is that because PLM is effectively housed in the National Department of Health, it's seen as an integrated partner. There's no restriction or firewall on shared information, access to information when it's available.* – SA Phase I

740. Governance	How governance structures enhance credibility and trust; accountability; hierarchy; meetings; overarching authority	
904. Great quote	Quotations that are particularly illustrative of particular themes and good for paper	
